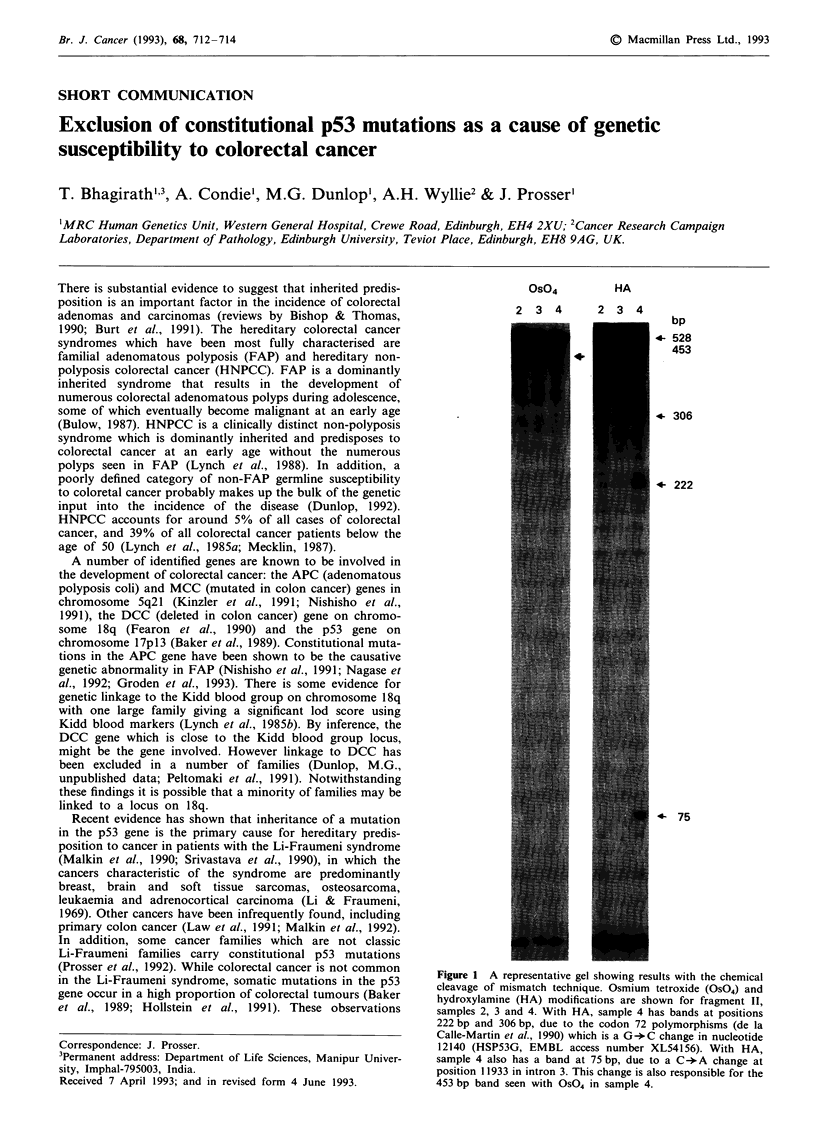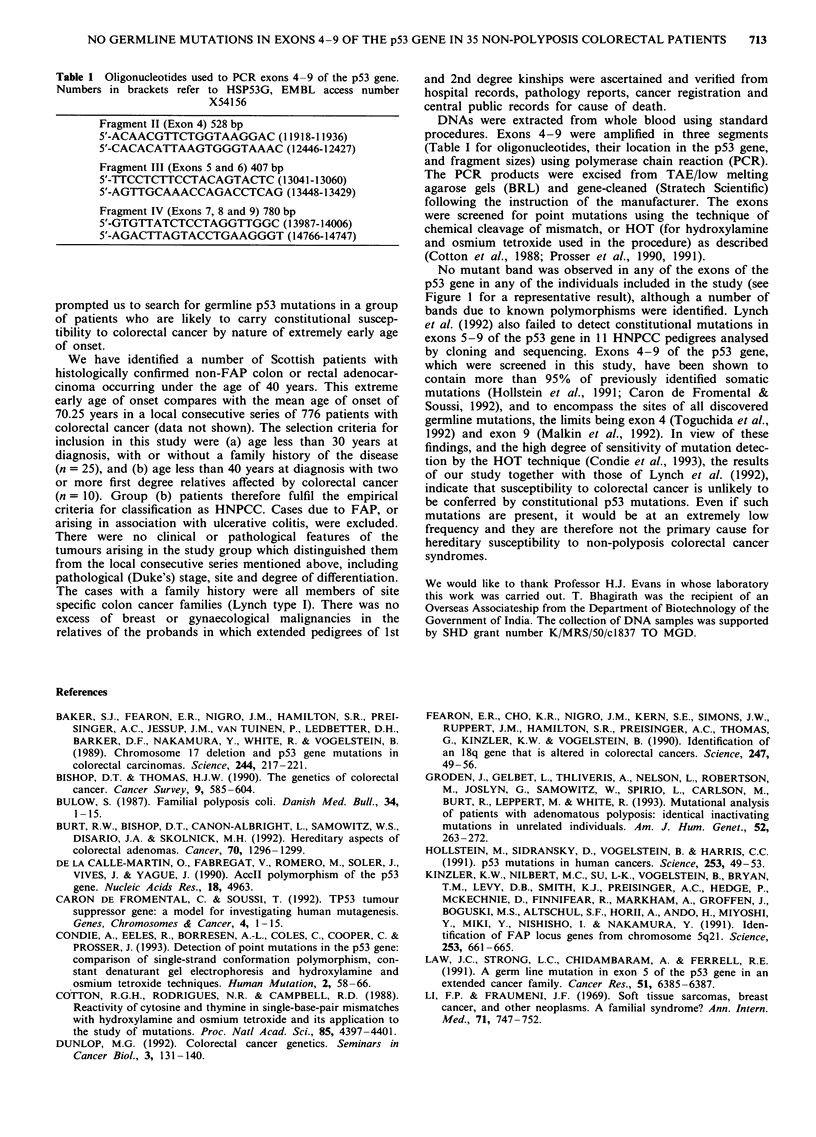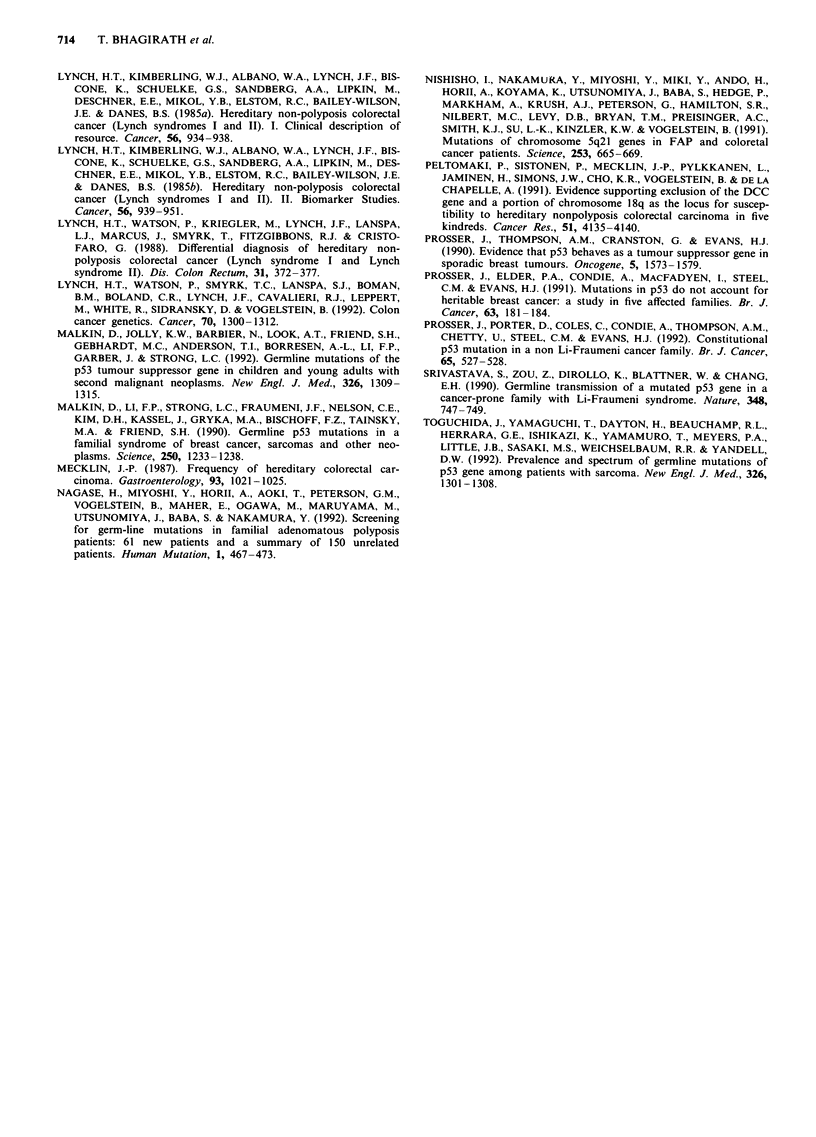# Exclusion of constitutional p53 mutations as a cause of genetic susceptibility to colorectal cancer.

**DOI:** 10.1038/bjc.1993.415

**Published:** 1993-10

**Authors:** T. Bhagirath, A. Condie, M. G. Dunlop, A. H. Wyllie, J. Prosser

**Affiliations:** MRC Human Genetics Unit, Western General Hospital, Edinburgh, UK.

## Abstract

**Images:**


 © Macmillan Press Ltd., 1993

SHORT COMMUNICATION

### Exclusion of constitutional p53 mutations as a cause of genetic susceptibility to colorectal cancer

T. Bhagirath[1,3], A. Condie[1], M.G. Dunlop[1], A.H. Wyllie[2] & J. Prosser[1]

[1]*MRC Human Genetics Unit, Western General Hospital, Crewe Road, Edinburgh, EH4 2XU;* [2]*Cancer Research Campaign Laboratories, Department of Pathology, Edinburgh University, Teviot Place, Edinburgh, EH8 9AG, UK.*

There is substantial evidence to suggest that inherited predisposition is an important factor in the incidence of colorectal adenomas and carcinomas (reviews by Bishop & Thomas, 1990; Burt *et al.*, 1991). The hereditary colorectal cancer syndromes which have been most fully characterised are familial adenomatous polyposis (FAP) and hereditary non-polyposis colorectal cancer (HNPCC). FAP is a dominantly inherited syndrome that results in the development of numerous colorectal adenomatous polyps during adolescence, some of which eventually become malignant at an early age (Bulow, 1987). HNPCC is a clinically distinct non-polyposis syndrome which is dominantly inherited and predisposes to colorectal cancer at an early age without the numerous polyps seen in FAP (Lynch *et al.*, 1988). In addition, a poorly defined category of non-FAP germline susceptibility to coloretal cancer probably makes up the bulk of the genetic input into the incidence of the disease (Dunlop, 1992). HNPCC accounts for around 5% of all cases of colorectal cancer, and 39% of all colorectal cancer patients below the age of 50 (Lynch *et al.*, 1985*a*; Mecklin, 1987).

A number of identified genes are known to be involved in the development of colorectal cancer: the APC (adenomatous polyposis coli) and MCC (mutated in colon cancer) genes in chromosome 5q21 (Kinzler *et al.*, 1991; Nishisho *et al.*, 1991), the DCC (deleted in colon cancer) gene on chromosome 18q (Fearon *et al.*, 1990) and the p53 gene on chromosome 17p13 (Baker *et al.*, 1989). Constitutional mutations in the APC gene have been shown to be the causative genetic abnormality in FAP (Nishisho *et al.*, 1991; Nagase *et al.*, 1992; Groden *et al.*, 1993). There is some evidence for genetic linkage to the Kidd blood group on chromosome 18q with one large family giving a significant lod score using Kidd blood markers (Lynch *et al.*, 1985*b*). By inference, the DCC gene which is close to the Kidd blood group locus, might be the gene involved. However linkage to DCC has been excluded in a number of families (Dunlop, M.G., unpublished data; Peltomaki *et al.*, 1991). Notwithstanding these findings it is possible that a minority of families may be linked to a locus on 18q.

Recent evidence has shown that inheritance of a mutation in the p53 gene is the primary cause for hereditary predisposition to cancer in patients with the Li-Fraumeni syndrome (Malkin *et al.*, 1990; Srivastava *et al.*, 1990), in which the cancers characteristic of the syndrome are predominantly breast, brain and soft tissue sarcomas, osteosarcoma, leukaemia and adrenocortical carcinoma (Li & Fraumeni, 1969). Other cancers have been infrequently found, including primary colon cancer (Law *et al.*, 1991; Malkin *et al.*, 1992). In addition, some cancer families which are not classic Li-Fraumeni families carry constitutional p53 mutations (Prosser *et al.*, 1992). While colorectal cancer is not common in the Li-Fraumeni syndrome, somatic mutations in the p53 gene occur in a high proportion of colorectal tumours (Baker *et al.*, 1989; Hollstein *et al.*, 1991). These observations

**Figure 1** A representative gel showing results with the chemical cleavage of mismatch technique. Osmium tetroxide ($OsO_4$) and hydroxylamine (HA) modifications are shown for fragment II, samples 2, 3 and 4. With HA, sample 4 has bands at positions 222 bp and 306 bp, due to the codon 72 polymorphisms (de la Calle-Martin *et al.*, 1990) which is a G→C change in nucleotide 12140 (HSP53G, EMBL access number XL54156). With HA, sample 4 also has a band at 75 bp, due to a C→A change at position 11933 in intron 3. This change is also responsible for the 453 bp band seen with $OsO_4$ in sample 4.

Correspondence: J. Prosser.
[3]Permanent address: Department of Life Sciences, Manipur University, Imphal-795003, India.
Received 7 April 1993; and in revised form 4 June 1993.

**Table 1** Oligonucleotides used to PCR exons 4–9 of the p53 gene. Numbers in brackets refer to HSP53G, EMBL access number X54156

| |
|---|
| Fragment II (Exon 4) 528 bp |
| 5'-ACAACGTTCTGGTAAGGAC (11918-11936) |
| 5'-CACACATTAAGTGGGTAAAC (12446-12427) |
| Fragment III (Exons 5 and 6) 407 bp |
| 5'-TTCCTCTTCCTACAGTACTC (13041-13060) |
| 5'-AGTTGCAAACCAGACCTCAG (13448-13429) |
| Fragment IV (Exons 7, 8 and 9) 780 bp |
| 5'-GTGTTATCTCCTAGGTTGGC (13987-14006) |
| 5'-AGACTTAGTACCTGAAGGGT (14766-14747) |

prompted us to search for germline p53 mutations in a group of patients who are likely to carry constitutional susceptibility to colorectal cancer by nature of extremely early age of onset.

We have identified a number of Scottish patients with histologically confirmed non-FAP colon or rectal adenocarcinoma occurring under the age of 40 years. This extreme early age of onset compares with the mean age of onset of 70.25 years in a local consecutive series of 776 patients with colorectal cancer (data not shown). The selection criteria for inclusion in this study were (a) age less than 30 years at diagnosis, with or without a family history of the disease ($n = 25$), and (b) age less than 40 years at diagnosis with two or more first degree relatives affected by colorectal cancer ($n = 10$). Group (b) patients therefore fulfil the empirical criteria for classification as HNPCC. Cases due to FAP, or arising in association with ulcerative colitis, were excluded. There were no clinical or pathological features of the tumours arising in the study group which distinguished them from the local consecutive series mentioned above, including pathological (Duke's) stage, site and degree of differentiation. The cases with a family history were all members of site specific colon cancer families (Lynch type I). There was no excess of breast or gynaecological malignancies in the relatives of the probands in which extended pedigrees of 1st and 2nd degree kinships were ascertained and verified from hospital records, pathology reports, cancer registration and central public records for cause of death.

DNAs were extracted from whole blood using standard procedures. Exons 4–9 were amplified in three segments (Table I for oligonucleotides, their location in the p53 gene, and fragment sizes) using polymerase chain reaction (PCR). The PCR products were excised from TAE/low melting agarose gels (BRL) and gene-cleaned (Stratech Scientific) following the instruction of the manufacturer. The exons were screened for point mutations using the technique of chemical cleavage of mismatch, or HOT (for hydroxylamine and osmium tetroxide used in the procedure) as described (Cotton *et al.*, 1988; Prosser *et al.*, 1990, 1991).

No mutant band was observed in any of the exons of the p53 gene in any of the individuals included in the study (see Figure 1 for a representative result), although a number of bands due to known polymorphisms were identified. Lynch *et al.* (1992) also failed to detect constitutional mutations in exons 5–9 of the p53 gene in 11 HNPCC pedigrees analysed by cloning and sequencing. Exons 4–9 of the p53 gene, which were screened in this study, have been shown to contain more than 95% of previously identified somatic mutations (Hollstein *et al.*, 1991; Caron de Fromental & Soussi, 1992), and to encompass the sites of all discovered germline mutations, the limits being exon 4 (Toguchida *et al.*, 1992) and exon 9 (Malkin *et al.*, 1992). In view of these findings, and the high degree of sensitivity of mutation detection by the HOT technique (Condie *et al.*, 1993), the results of our study together with those of Lynch *et al.* (1992), indicate that susceptibility to colorectal cancer is unlikely to be conferred by constitutional p53 mutations. Even if such mutations are present, it would be at an extremely low frequency and they are therefore not the primary cause for hereditary susceptibility to non-polyposis colorectal cancer syndromes.

We would like to thank Professor H.J. Evans in whose laboratory this work was carried out. T. Bhagirath was the recipient of an Overseas Associateship from the Department of Biotechnology of the Government of India. The collection of DNA samples was supported by SHD grant number K/MRS/50/c1837 TO MGD.

### References

BAKER, S.J., FEARON, E.R., NIGRO, J.M., HAMILTON, S.R., PREISINGER, A.C., JESSUP, J.M., VAN TUINEN, P., LEDBETTER, D.H., BARKER, D.F., NAKAMURA, Y., WHITE, R. & VOGELSTEIN, B. (1989). Chromosome 17 deletion and p53 gene mutations in colorectal carcinomas. *Science*, **244**, 217–221.

BISHOP, D.T. & THOMAS, H.J.W. (1990). The genetics of colorectal cancer. *Cancer Survey*, **9**, 585–604.

BULOW, S. (1987). Familial polyposis coli. *Danish Med. Bull.*, **34**, 1–15.

BURT, R.W., BISHOP, D.T., CANON-ALBRIGHT, L., SAMOWITZ, W.S., DISARIO, J.A. & SKOLNICK, M.H. (1992). Hereditary aspects of colorectal adenomas. *Cancer*, **70**, 1296–1299.

DE LA CALLE-MARTIN, O., FABREGAT, V., ROMERO, M., SOLER, J., VIVES, J. & YAGUE, J. (1990). AccII polymorphism of the p53 gene. *Nucleic Acids Res.*, **18**, 4963.

CARON DE FROMENTAL, C. & SOUSSI, T. (1992). TP53 tumour suppressor gene: a model for investigating human mutagenesis. *Genes, Chromosomes & Cancer*, **4**, 1–15.

CONDIE, A., EELES, R., BORRESEN, A.-L., COLES, C., COOPER, C. & PROSSER, J. (1993). Detection of point mutations in the p53 gene: comparison of single-strand conformation polymorphism, constant denaturant gel electrophoresis and hydroxylamine and osmium tetroxide techniques. *Human Mutation*, **2**, 58–66.

COTTON, R.G.H., RODRIGUES, N.R. & CAMPBELL, R.D. (1988). Reactivity of cytosine and thymine in single-base-pair mismatches with hydroxylamine and osmium tetroxide and its application to the study of mutations. *Proc. Natl Acad. Sci.*, **85**, 4397–4401.

DUNLOP, M.G. (1992). Colorectal cancer genetics. *Seminars in Cancer Biol.*, **3**, 131–140.

FEARON, E.R., CHO, K.R., NIGRO, J.M., KERN, S.E., SIMONS, J.W., RUPPERT, J.M., HAMILTON, S.R., PREISINGER, A.C., THOMAS, G., KINZLER, K.W. & VOGELSTEIN, B. (1990). Identification of an 18q gene that is altered in colorectal cancers. *Science*, **247**, 49–56.

GRODEN, J., GELBET, L., THLIVERIS, A., NELSON, L., ROBERTSON, M., JOSLYN, G., SAMOWITZ, W., SPIRIO, L., CARLSON, M., BURT, R., LEPPERT, M. & WHITE, R. (1993). Mutational analysis of patients with adenomatous polyposis: identical inactivating mutations in unrelated individuals. *Am. J. Hum. Genet.*, **52**, 263–272.

HOLLSTEIN, M., SIDRANSKY, D., VOGELSTEIN, B. & HARRIS, C.C. (1991). p53 mutations in human cancers. *Science*, **253**, 49–53.

KINZLER, K.W., NILBERT, M.C., SU, L-K., VOGELSTEIN, B., BRYAN, T.M., LEVY, D.B., SMITH, K.J., PREISINGER, A.C., HEDGE, P., MCKECHNIE, D., FINNIFEAR, R., MARKHAM, A., GROFFEN, J., BOGUSKI, M.S., ALTSCHUL, S.F., HORII, A., ANDO, H., MIYOSHI, Y., MIKI, Y., NISHISHO, I. & NAKAMURA, Y. (1991). Identification of FAP locus genes from chromosome 5q21. *Science*, **253**, 661–665.

LAW, J.C., STRONG, L.C., CHIDAMBARAM, A. & FERRELL, R.E. (1991). A germ line mutation in exon 5 of the p53 gene in an extended cancer family. *Cancer Res.*, **51**, 6385–6387.

LI, F.P. & FRAUMENI, J.F. (1969). Soft tissue sarcomas, breast cancer, and other neoplasms. A familial syndrome? *Ann. Intern. Med.*, **71**, 747–752.

LYNCH, H.T., KIMBERLING, W.J., ALBANO, W.A., LYNCH, J.F., BISCONE, K., SCHUELKE, G.S., SANDBERG, A.A., LIPKIN, M., DESCHNER, E.E., MIKOL, Y.B., ELSTOM, R.C., BAILEY-WILSON, J.E. & DANES, B.S. (1985*a*). Hereditary non-polyposis colorectal cancer (Lynch syndromes I and II). I. Clinical description of resource. *Cancer*, **56**, 934–938.

LYNCH, H.T., KIMBERLING, W.J., ALBANO, W.A., LYNCH, J.F., BISCONE, K., SCHUELKE, G.S., SANDBERG, A.A., LIPKIN, M., DESCHNER, E.E., MIKOL, Y.B., ELSTOM, R.C., BAILEY-WILSON, J.E. & DANES, B.S. (1985*b*). Hereditary non-polyposis colorectal cancer (Lynch syndromes I and II). II. Biomarker Studies. *Cancer*, **56**, 939–951.

LYNCH, H.T., WATSON, P., KRIEGLER, M., LYNCH, J.F., LANSPA, L.J., MARCUS, J., SMYRK, T., FITZGIBBONS, R.J. & CRISTOFARO, G. (1988). Differential diagnosis of hereditary non-polyposis colorectal cancer (Lynch syndrome I and Lynch syndrome II). *Dis. Colon Rectum*, **31**, 372–377.

LYNCH, H.T., WATSON, P., SMYRK, T.C., LANSPA, S.J., BOMAN, B.M., BOLAND, C.R., LYNCH, J.F., CAVALIERI, R.J., LEPPERT, M., WHITE, R., SIDRANSKY, D. & VOGELSTEIN, B. (1992). Colon cancer genetics. *Cancer*, **70**, 1300–1312.

MALKIN, D., JOLLY, K.W., BARBIER, N., LOOK, A.T., FRIEND, S.H., GEBHARDT, M.C., ANDERSON, T.I., BORRESEN, A.-L., LI, F.P., GARBER, J. & STRONG, L.C. (1992). Germline mutations of the p53 tumour suppressor gene in children and young adults with second malignant neoplasms. *New Engl. J. Med.*, **326**, 1309–1315.

MALKIN, D., LI, F.P., STRONG, L.C., FRAUMENI, J.F., NELSON, C.E., KIM, D.H., KASSEL, J., GRYKA, M.A., BISCHOFF, F.Z., TAINSKY, M.A. & FRIEND, S.H. (1990). Germline p53 mutations in a familial syndrome of breast cancer, sarcomas and other neoplasms. *Science*, **250**, 1233–1238.

MECKLIN, J.-P. (1987). Frequency of hereditary colorectal carcinoma. *Gastroenterology*, **93**, 1021–1025.

NAGASE, H., MIYOSHI, Y., HORII, A., AOKI, T., PETERSON, G.M., VOGELSTEIN, B., MAHER, E., OGAWA, M., MARUYAMA, M., UTSUNOMIYA, J., BABA, S. & NAKAMURA, Y. (1992). Screening for germ-line mutations in familial adenomatous polyposis patients: 61 new patients and a summary of 150 unrelated patients. *Human Mutation*, **1**, 467–473.

NISHISHO, I., NAKAMURA, Y., MIYOSHI, Y., MIKI, Y., ANDO, H., HORII, A., KOYAMA, K., UTSUNOMIYA, J., BABA, S., HEDGE, P., MARKHAM, A., KRUSH, A.J., PETERSON, G., HAMILTON, S.R., NILBERT, M.C., LEVY, D.B., BRYAN, T.M., PREISINGER, A.C., SMITH, K.J., SU, L.-K., KINZLER, K.W. & VOGELSTEIN, B. (1991). Mutations of chromosome 5q21 genes in FAP and coloretal cancer patients. *Science*, **253**, 665–669.

PELTOMAKI, P., SISTONEN, P., MECKLIN, J.-P., PYLKKANEN, L., JAMINEN, H., SIMONS, J.W., CHO, K.R., VOGELSTEIN, B. & DE LA CHAPELLE, A. (1991). Evidence supporting exclusion of the DCC gene and a portion of chromosome 18q as the locus for susceptibility to hereditary nonpolyposis colorectal carcinoma in five kindreds. *Cancer Res.*, **51**, 4135–4140.

PROSSER, J., THOMPSON, A.M., CRANSTON, G. & EVANS, H.J. (1990). Evidence that p53 behaves as a tumour suppressor gene in sporadic breast tumours. *Oncogene*, **5**, 1573–1579.

PROSSER, J., ELDER, P.A., CONDIE, A., MACFADYEN, I., STEEL, C.M. & EVANS, H.J. (1991). Mutations in p53 do not account for heritable breast cancer: a study in five affected families. *Br. J. Cancer*, **63**, 181–184.

PROSSER, J., PORTER, D., COLES, C., CONDIE, A., THOMPSON, A.M., CHETTY, U., STEEL, C.M. & EVANS, H.J. (1992). Constitutional p53 mutation in a non Li-Fraumeni cancer family. *Br. J. Cancer*, **65**, 527–528.

SRIVASTAVA, S., ZOU, Z., DIROLLO, K., BLATTNER, W. & CHANG, E.H. (1990). Germline transmission of a mutated p53 gene in a cancer-prone family with Li-Fraumeni syndrome. *Nature*, **348**, 747–749.

TOGUCHIDA, J., YAMAGUCHI, T., DAYTON, H., BEAUCHAMP, R.L., HERRARA, G.E., ISHIKAZI, K., YAMAMURO, T., MEYERS, P.A., LITTLE, J.B., SASAKI, M.S., WEICHSELBAUM, R.R. & YANDELL, D.W. (1992). Prevalence and spectrum of germline mutations of p53 gene among patients with sarcoma. *New Engl. J. Med.*, **326**, 1301–1308.